# The Use of Low-Dose Recombinant Tissue Plasminogen Activator to Treat a Preterm Infant with an Intrauterine Spontaneous Arterial Thromboembolism

**DOI:** 10.4274/tjh.2015.0016

**Published:** 2015-12-03

**Authors:** Yaşar Demirelli, Kadir Şerafettin Tekgündüz, İbrahim Caner, Mustafa Kara

**Affiliations:** 1 Atatürk University Faculty of Medicine, Division of Neonatology, Erzurum, Turkey

**Keywords:** Preterm, Thromboembolism, tissue, Plasminogen, Intrauterine arterial thromboembolism, Low dose recombinant tPA therapy

## Abstract

Neonatal thromboembolic events are rare, and only a few cases of intrauterine spontaneous arterial thromboembolisms have been reported in the literature. Thrombolytic therapy with recombinant tissue plasminogen activator is usually the preferred treatment because it has a short half-life, fewer systemic side effects, and a strong, specific affinity for fibrin. Protocols vary from center to center, but there is still no consensus regarding the proper dosage or treatment duration. Herein, we present the case of an intrauterine spontaneous arterial thromboembolism in a preterm infant that completely resolved after being treated with low-dose recombinant tissue plasminogen activator (0.02 mg/kg/h).

## Abstract (tr)

Yenidoğanda tromboembolik olaylar sık değildir. Literatürde spontan intrauterin arteriyel tromboz olgusu oldukça az bildirilmiştir. Tromboemboli tedavisinde yarılanma ömrü kısa olduğu için rekombinan doku plazminojen aktivatörü çoğunlukla tercih edilmektedir. Tedavi protokolü merkezden merkeze değişiklik göstermesine karşın, doz ve süre konusunda bir uzlaşı yoktur. Biz burada kullanılabilecek en düşük dozlardan biriyle (0,02 mg/kg/doz) tamamen iyileşme sağladığımız intrauterin spontan arteriyel trombozlu bir olguyu sunmak istedik.

## INTRODUCTION

Spontaneous arterial thromboembolisms are a serious cause of mortality and morbidity in the neonatal period, and the congenital, acquired, and inherited prothrombotic states of this condition along with maternal characteristics have been identified as significant risk factors. The hypofibrinolytic situation in neonates, especially in premature infants, includes hemodynamic changes during the transition from the fetal period to the neonatal period which may predispose infants to these types of thromboembolisms [1]. The goal of treatment is to prevent life-threatening situations that might occur because of the embolism and the recurrence of thrombosis while also minimizing the risk of bleeding. Generally, recombinant tissue plasminogen activator (rtPA) is the first choice of treatment because it is nonantigenic, has a short half-life, produces rapidly reversible hypocoagulability, and possesses a strong, specific affinity for fibrin, but there is no consensus regarding the proper dosage or treatment duration [2]. In this study, we present the case of a premature baby with an intrauterine spontaneous arterial thromboembolism which developed at the level of the left brachial artery and was successfully treated with low-dose rtPA for a short period of time.

## CASE PRESENTATION

**Figure 1:**
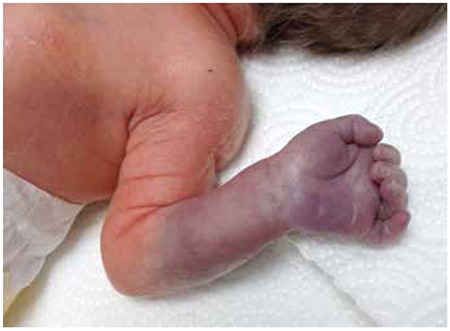
View of the patient’s left forearm from the elbow to the fingertips at birth. Note the pale, cyanotic appearance.

**Figure 2:**
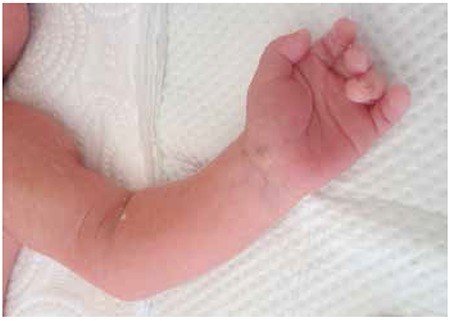
The patient experienced a complete recovery four hours after the recombinant tissue plasminogen activator infusion.

A male baby (1,530 g) was born at the 32nd gestational week via an emergency caesarean section because of anhydroamnios and vaginal bleeding. The mother had not had prenatal care. The mother was 24 years old, and neither the mother nor the infant had any complications during the surgical procedure. The Apgar scores at one and five minutes were 6 and 9 respectively, and the cord blood pH was normal. In addition, there was no history of maternal diabetes nor preeclampsia. However, the baby did receive a single dose of surfactant due to the presence of mild respiratory distress syndrome. The patient’s left forearm from the elbow to the fingertips had a pale and cyanotic appearance at birth that persisted afterwards (Figure 1), but the baby’s vital signs were normal. No brachial arterial flow was detected from the level of the elbow nor was there any distal radioulnar flow on Doppler ultrasonography (USG). Furthermore, no cardiac pathology was detected on an echocardiographic examination. The blood count was normal, there was no polycythemia or thrombocytopenia, and the coagulometer readings and fibrinogen values were also within normal ranges. After obtaining the informed consent of the infant’s parents, low-dose (0.02 mg/kg/h) rtPA (alteplase) was administered along with low-molecular-weight heparin (LMWH) (Clexane® 4000 IU/0.4 mL; 100 IU/kg/dose twice daily) in the first hour after birth. The transfontanelle USG was normal before and after the rtPA infusion, and distal pulses were detected by Doppler USG at the fourth hour of infusion (Figure 2). Hence, the rtPA was discontinued although the LMWH continued to be administered for six additional weeks. At follow-up, no complications or thrombosis had developed, and the screening for inherited thrombophilias [factor V Leiden mutation, homozygous protein C and S deficiency, prothrombin G20210A mutation, methyltetrahydrofolate reductase (MTHFR) gene mutation, antithrombin III, factor 12, anticardiolipin antibodies, homocysteine, and lipoprotein (a)] was normal.

## DISCUSSION AND REVIEW OF THE LITERATURE

The incidence of clinically apparent neonatal thrombosis in recent reports has varied from 5.1 per 100,000 births to 2.4 per 1,000 admissions [3,4]. Most cases of thromboembolism during the neonatal period are due to vascular interventions; however, there have been very few reported cases of intrauterine spontaneous arterial thromboembolism in the literature [5]. Various risk factors, such as being an infant of a diabetic mother, having polycythemia, dehydration, sepsis, asphyxia, or oligohydroamnios, and being of the male gender can contribute to this condition, but the pathophysiology has not yet been fully clarified [1,6]. In the case of our patient, we observed cyanosis in the left forearm at birth.

Saracco et al. reported that prepartum risk factors, including emergency caesarean sections, are significantly associated with arterial ischemic stroke in neonates, and Rashish et al. identified decreased fetal movements, oligohydramnios, preeclampsia, and maternal diabetes as maternal risk factors [1,6]. In addition, they also found that when oligohydramnios is present, decreased fetal movement may cause venous stasis and thrombus formation. We believe that the caesarean delivery and anhydroamnios were the primary risk factors in our patient, but we could not determine whether the thromboembolism occured within the uterus or during the birth process. However, the apparent lack of necrosis in the forearm at birth suggests that the thromboembolism occurred just prior to delivery.

Rashish et al. hypothesized that spontaneous arterial thromboembolisms originate in utero and develop secondary to placental-fetal umblical pathology [1]. Furthermore, they reported that the most common site of thromboembolisms were, in order of frequency, the umblical artery, the aorta, and the extremities. In our patient, the left forearm was the site of the thromboembolism. However, no thrombophilic state was detected in our patient, which might have been because of intrauterine pathology.

In newborn infants, appropriate, adequate, timely intervention of thromboembolisms is very important in order to reduce morbidity and mortality. In the literature, there are several studies that have reported the use of streptokinase, urokinase, and rtPA for thrombolytic therapy, and in recent years the popularity of rtPA has been on the rise because of its short half-life, nonantigenic qualities, and locally specific action on plasminogen-bound fibrin [7,8]. In addition, it also has fewer systemic side effects than other agents used in thrombolytic therapy [9,10]. However, this type of therapy is associated with significant bleeding complications such as intracranial hemorrhage, and Monagle et al. determined that the most frequent problem was bleeding at the sites of invasive procedures that required treatment with blood products [11]. Furthermore, they also found a connection between prolonged thrombolytic infusion and increased bleeding. In our case the short duration of rtPA treatment, which was limited to four hours, may have played a role in the prevention of complications. However, we did inform the patient’s parents about the possible complications before initiating the treatment.

Many case series have been reported on the use of low and high dose rtPa both with and without a bolus as well as in conjuction with other anticoagulant agents, and there is general agreement that rtPa is safe and effective. There is still no consensus concerning the correct dosage and duration of treatment [3,5,12]. Olgun et al. started rtPa 13 of their 22 patients (range 5 days-17 years old) with extremity or cardiac thrombosis on low-dose treatment (0.01-0.03 mg/kg/h), and in six of these the dosage was increased over the course of the treatment [12]. Their findings showed that seven patients experienced complete recovery within 4 to 36 hours and that no significant complications were seen except for bleeding at the vascular puncture site in two patients. However, five patients with fibrinogen deficiency in the high-dose group reported epistaxis and melena. In our patient, we used low-dose rtPA (0.02 mg/kg/h) and observed a complete recovery within four hours after the infusion without any complications. We administered rtPA (alteplase) along with LMWH in the first hour after birth, and this treatment has previously been reported to be safe and effective, especially for preventing second thrombi [13].

In conclusion, low-dose rtPA proved to be successful for the treatment of arterial thromboembolism in our patient. However, a randomized prospective study is needed to determine the precise dosage and duration of this treatment in premature newborns.
